# Triggering of Parkin Mitochondrial Translocation in Mitophagy: Implications for Liver Diseases

**DOI:** 10.3389/fphar.2016.00100

**Published:** 2016-04-29

**Authors:** Nabil Eid, Yuko Ito, Yoshinori Otsuki

**Affiliations:** ^1^Department of Anatomy and Cell Biology, Division of Life Sciences, Osaka Medical CollegeOsaka, Japan; ^2^Osaka Medical CollegeOsaka, Japan

**Keywords:** ethanol, liver, lipophagy, mitophagy, Parkin, spheroids, 8-OHdG

## Abstract

A growing body of evidence based on *in vitro* studies indicates that mitophagy (selective autophagic clearance of damaged mitochondria) is a prosurvival mechanism associated with cellular exposure to various mitochondrial stressors. Very recently, a limited number of publications on animal-based models of alcoholic fatty liver diseases have reported that Parkin-mediated mitophagy may mitigate hepatocyte apoptosis, improve mitochondrial quality and suppress steatosis (lipid accumulation). From this perspective, the authors focus on the mechanisms of Parkin mitochondrial translocation (a key consideration in mitophagy activation) and therapeutic implications of mitophagy in liver disease. DNA repair and other functions of Parkin beyond mitophagy are also briefly discussed. The paper additionally shows original data from the authors’ current research indicating enhanced hepatic mitophagy in ethanol-treated rats, which is associated with Parkin mitochondrial translocation triggered by oxidative mitochondrial DNA damage. Natural or pharmaceutical products that may trigger Parkin mitochondrial translocation in hepatocytes and/or suppress repressors of such translocation could be a potential therapeutic target in alcoholic and non-alcoholic fatty liver disease.

## The Pink1–Parkin Pathway in Mitophagy

Autophagy (macroautophagy) is a prosurvival pathway for lysosomal degradation of most cellular components in response to diverse conditions of stress, such as oxidative stress, DNA damage, and lipid overload. Selective autophagic elimination of proapoptotic mitochondria is called mitophagy (Eid et al., 2013b; Lemasters, 2014). The PINK1/Parkin pathway involves the interplay of two recessive Parkinson’s-linked genes [PTEN-induced kinase 1 (PINK1) and Parkin (an E3 ubiquitin ligase)], which maintain mitochondrial homeostasis and clear dysfunctional mitochondria via mitophagy. Mutations affecting PINK1–Parkin genes cause Parkinson’s disease (PD; a neurodegenerative illness characterized by accumulation of dysfunctional mitochondria). In mammals, various effectors (including the mitophagy receptors NIX and BNIP3 and the PINK1–Parkin pathway) contribute to the elimination of damaged mitochondria under exposure to mitochondrial damaging agents (Youle and Narendra, 2011; Amadoro et al., 2014).

In healthy mammalian cells, the mitochondrial level of PINK1 is very low, while Parkin normally resides in the cytoplasm (Youle and Narendra, 2011; Eid et al., 2013a; Amadoro et al., 2014). Mitophagy is initiated by accumulation of PINK1 at the outer membrane of damaged mitochondria, resulting in the recruitment of cytoplasmic Parkin to those mitochondria. The PINK1–Parkin interaction in damaged mitochondria promotes mitophagy through protein ubiquitination and subsequent mitochondrial fragmentation and engulfment of mitochondria by LC3-mediated autophagosomes forming mitophagosomes. The latter fuse with lysosomes forming mitophagolysosomes with specific perinuclear localization (Eid et al., 2013a; Kim et al., 2013; Amadoro et al., 2014; Lemasters, 2014). Based mostly on *in vitro* studies, it is considered that various mechanisms (such as oxidative stress and mitochondrial depolarization/fission) and mitochondrial DNA (mtDNA) damage may activate mitophagy (Amadoro et al., 2014; Schneider and Cuervo, 2014; Zhang, 2015). Importantly, in consideration of similar mechanisms in various animal-based models, acute and chronic ethanol consumption has recently been reported to stimulate hepatic mitophagy resulting in reduction of steatosis and apoptosis (Ding et al., 2010; Eid et al., 2013a,b; Lin et al., 2013; Zhong et al., 2014; Williams et al., 2015).

## Ethanol-Induced Hepatic Mitophagy is Associated with Parkin Mitochondrial Translocation Triggered by Oxidative DNA Damage

**FIGURE 1:**
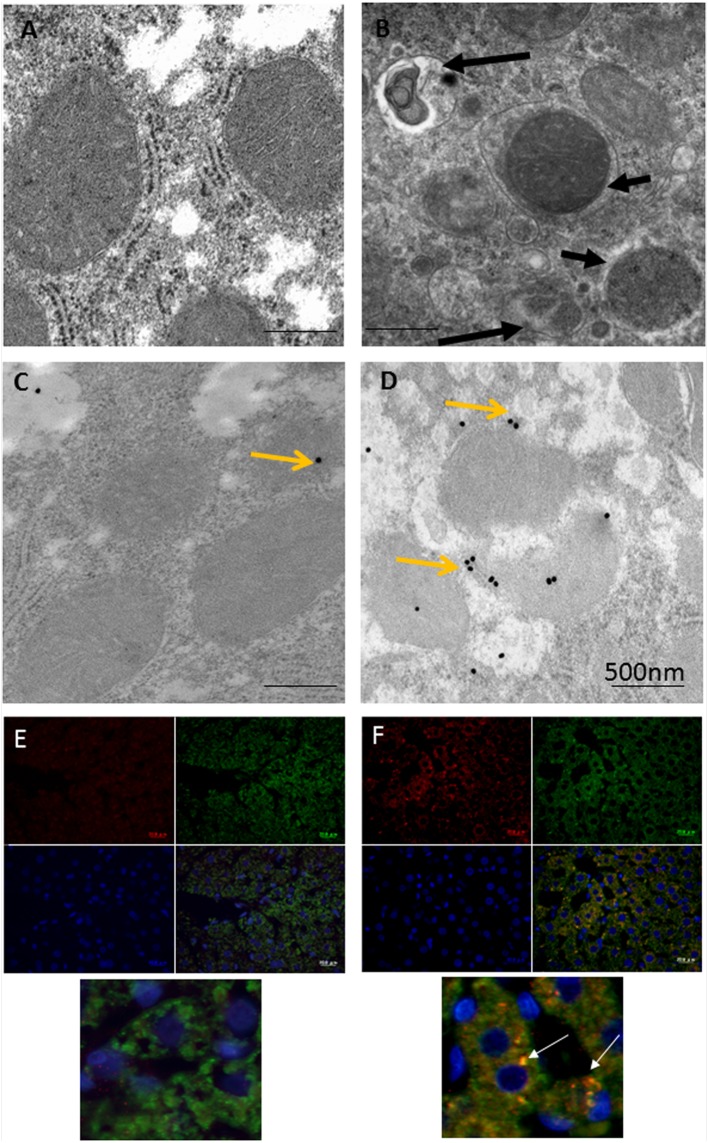
**Enhanced mitophagy in hepatocytes of ETRs. (A,B)** TEM of control **(A)** and ETRs **(B)**. The short black arrows indicate mitophagosomes, while the long black arrows show mitophagolysosomes. **(C,D)** IEM of Parkin in control **(C)** and ETRs **(D)**. Yellow arrows indicate 25 nm Parkin immunogold particles. **(E,F)** Immunofluorescence double labeling of Parkin (Red) and cytochrome c (green) in control **(E)** and ETRs **(F)**. The white arrows show colocalization signals on merging (yellow) in magnified areas below. Note that DAPI (blue) is for nuclear counterstaining.

**FIGURE 2:**
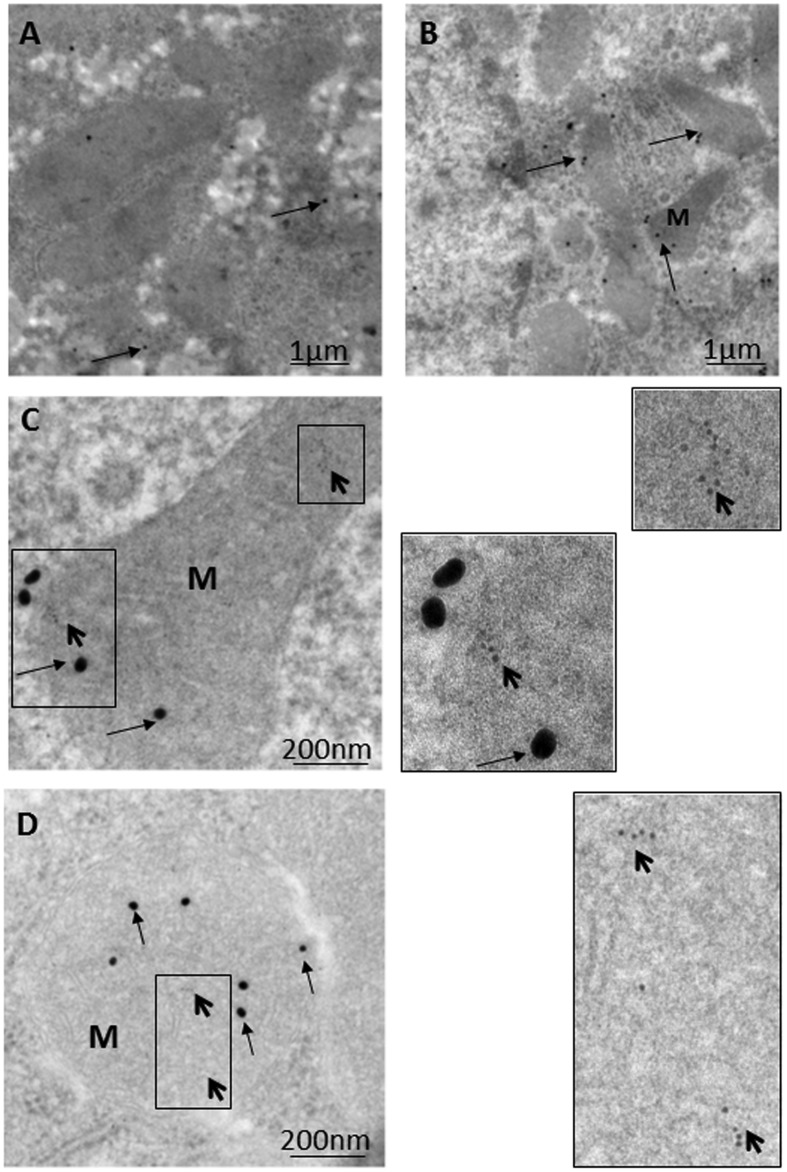
**Parkin translocation to mitochondria and mitophagosomes with accumulated 8-OHdG in hepatocytes of ETRs. (A)** Control while **(B–D)**, ETRs. The boxed areas in **C** and **D** are magnified on the right. The long and short arrows indicate Parkin (25 nm) and 8-OHdG (6 nm) immunogold particles, respectively. M, mitochondrion. The method of post-embedding immunogold double labeling of Parkin (NB100-91921) and 8-OHdG (N45.1) was performed according to the manufacturer’s protocols (Aurion, Wageningen, Netherlands; http://www.aurion.nl/products/gold_sols.php) and recent publications (Eid et al., 2013a, 2016).

Immunoelectron microscopy (IEM) techniques play important roles in the exploration of components and trafficking in autophagic machinery, especially in relation to membranous structures that can only be clearly identified and localized using electron microscopes (Eskelinen et al., 2011; Lenzi et al., 2012; Eid et al., 2013a). As most research on Parkin-related mitophagy has involved the examination of cultured neuronal cells with focus on downstream signaling events in mitophagy, upstream signaling pathways remain comparatively poorly characterized (Grenier et al., 2013). Based on various light and electron microscopic techniques, the authors recently investigated the mechanism behind the triggering of Parkin mitochondrial translocation and mitophagy induction using a model rat liver with exposure to ethanol binge conditions (Nogales et al., 2014; Eid et al., 2016). As shown in **Figure 1**, compared to the control hepatocyte (**Figure 1A**), a marked increase in the number of mitophagic vacuoles (mitophagosomes and mitophagolysosomes; **Figure 1B**) was observed in the majority of normal hepatocytes in ethanol-treated rats (ETRs) within 6 h of a single intraperitoneal injection of ethanol (5 g/kg). However, the level of apoptotic hepatocytes was very low (data not shown). This enhanced mitophagy of ETRs hepatocytes was associated Parkin mitochondrial translocation as shown by IEM (**Figures 1C,D**) and immunofluorescence double labeling of Parkin and cytochrome c (**Figures 1E,F**). Also, Parkin also was detected clearly in mitophagosomes as shown in **Supplementary Figure S1**. Interestingly in ETRs, and as a novel unreported finding revealed by IEM, Parkin was observed to be selectively translocated to hepatocyte mitochondria and mitophagosomes enriched with 8-OHdG, which is a marker of oxidative mtDNA damage and mutagenicity (Gao et al., 2004; Guo et al., 2008) (**Figure 2**). We confirmed this colocalization of Parkin and 8-OHdG using immunofluorescence double labeling technique (Eid et al., 2016). Moreover, as shown in the control hepatocytes, Parkin expression was low and cytoplasmic, while the expression of 8-OHdG was very weak. Accordingly, consideration is required to determine the significance of Parkin co-localization with accumulated 8-OHdG in hepatocyte mitochondria of ETRs. In particular, there is a need for further research to establish whether there is simply a relation to the activation of cytoprotective mitophagy or whether Parkin serves other functions beyond mitophagy.

## Parkin Co-Localization with 8-OHdG in ETR Hepatocytes: Relevance to Mitophagy

Parkin co-localization with accumulated 8-OHdG in hepatocyte mitochondria of ETRs (**Figure 2**) may be a signal for mitophagy induction and formation of mitophagosomes via the triggering of Parkin mitochondrial translocation (Youle and Narendra, 2011; Zhang, 2015; Eid et al., 2016). This may be supported by the study of Gmitterová et al. (2009), who reported an increase in 8-OHdG levels in the brain and peripheral tissues of PD patients, where Parkin mutations are common (Youle and Narendra, 2011; Amadoro et al., 2014). In addition, the co-localization of Parkin and 8-OHdG may represent an ideal method for monitoring mitophagy compared with other methods involving Parkin co-localization with outer mitochondrial proteins because the latter proteins could be degraded by proteasome rather than mitophagy (Yoshii et al., 2011).

## Possible Functions of Parkin Beyond Mitophagy

Parkin co-localization with accumulated 8-OHdG in hepatocyte mitochondria of ETRs may be a stimulus for DNA repair and prevention of oncogenesis, as endogenous Parkin has a reported physical association with mtDNA (Rothfuss et al., 2009) and translocates to nuclei in cultured neuronal cells affected by oxidative DNA damage (Kao, 2009). Recent studies have also revealed further functions of mitochondrial Parkin in cell lines exposed to various stressors, including the suppression of mitochondrial spheroid formation (Yin and Ding, 2013; Eid et al., 2015; Khalil et al., 2015) and enhancement of mitochondrial-derived vesicle formation under oxidative stress (McLelland et al., 2014), stimulation of the selective escape of antiapoptotic proteins from mitochondria to the endoplasmic reticulum during mitophagy in stressed cells (Saita et al., 2013), and donation of mitochondrial-derived autophagosomal membranes in drug-treated breast cancer cells (Cook et al., 2014). Further studies in animal models of fatty liver disease are needed to investigate these functions of Parkin beyond mitophagy.

## Pharmacological Manipulation of Parkin as a Potential Therapeutic Target in Fatty Liver Disease

A cumulative body of evidence indicates that the mitochondrion is the main target for alcohol toxicity (Hoek et al., 2002). Accordingly, the use of autophagy and/or mitophagy inducers may represent a suitable strategy for improving mitochondrial function in alcoholic fatty liver (AFL) and non-alcoholic fatty liver (NAFL) disease. The activation of autophagy through mTOR inhibitor rapamycin has been shown to enhance autophagic removal of damaged mitochondria in PD (Siddiqui et al., 2012). Lin et al. (2013) found that pharmacological promotion of autophagy by carbamazepine or rapamycin enhanced lipophagy and possibly mitophagy in animal models of AFL and NAFL disease, and that it subsequently alleviated steatosis and hepatocyte damage. Natural or pharmacological stimulation of mitophagy via the upregulation of Parkin expression and/or its mitochondrial translocation may represent a promising therapeutic target in relation to AFL and NAFL disease. The specific transcription factors that may upregulate Parkin expression in ETR hepatocytes may be linked to the FOXO3a signaling pathway, as FOXO3a has been reported to enhance the expression of LC3 in hepatocytes of ethanol-treated mice (Ni et al., 2013) and to stimulate PINK1–Parkin-mediated mitophagy with grape-derived antioxidant in stressed heart tissue (Das et al., 2014). The results of a recent study (Yu et al., 2016) indicated that quercetin suppressed chronic ethanol-induced hepatic mitochondrial damage in mice by activating mitophagy via Parkin overexpression, which was mediated by increased nuclear translocation of FOXO3a. As recently reported by the authors of this paper, PINK1 overexpression on hepatocyte mitochondrial outer membranes of acute and chronic ETRs may be a major sensor for Parkin mitochondrial translocation and recognition of damaged mitochondria by autophagic machinery (Eid et al., 2013a, 2015, 2016). Meanwhile, it has also been found that Parkin mitochondrial translocation may be repressed by cytoplasmic P53, thus preventing mitophagy induction in the myocardial muscle of stressed mice (Hoshino et al., 2013). Suppression of Parkin mitochondrial translocation repressors may therefore stimulate mitophagy and could be of therapeutic importance in hepatosteatosis. Treatment of *ob/ob* mice (a genetic model for NAFL) with metformin was found to enhance prosurvival mitophagy in hepatocytes by suppressing the inhibitory interaction of cytosolic p53 with Parkin, allowing Parkin mitochondrial translocation and increasing the degradation of mitofusins (Song et al., 2016). However, certain precautions should be considered in relation to the stimulation of mitophagy in AFL associated with viral infection because some studies have found that the hepatitis C virus may induce mitophagy in hepatocytes as a prosurvival mechanism, conferring protection and stimulating viral multiplication (Osna et al., 2011; Kim et al., 2013).

## Conclusion

The proper understanding of the molecular mechanisms of mitophagy may be essential for the treatment of fatty liver disease induced by or associated with mitochondrial damage. IEM may be a powerful tool for detecting changes in subcellular localization of mitophagy proteins under various conditions, which may have diagnostic and therapeutic implications. Selective stimulation of Parkin-mediated mitophagy via the enhancement of its expression and/or mitochondrial translocation using natural or pharmaceutical products may have therapeutic potential for improving mitochondrial quality and survival, suppressing steatosis and preventing mutagenicity in fatty liver disease.

## Ethics Statement

The animals were maintained and treated according to the guidelines set by the Experimental Animal Research Committee of Osaka Medical College.

## Author Contributions

NE performed the experimental work, electron microscopic studies, and wrote the manuscript, YI participated in experimental work and design, YO participated in design of experiment and revised the paper.

## Conflict of Interest Statement

The authors declare that the research was conducted in the absence of any commercial or financial relationships that could be construed as a potential conflict of interest.

## Supplementary material

FIGURE S1 **Immunoelectron microscopy (IEM) of Parkin in mitophagosome of ethanol-treated rat (ETR) hepatocytes.** Black arrow indicate mitophagosome. White arrows in the inset indicate 6 nm Parkin immunogold particles. M, mitochondrion.
